# Relationships between the Foot Posture Index and foot kinematics during gait in individuals with and without patellofemoral pain syndrome

**DOI:** 10.1186/1757-1146-4-10

**Published:** 2011-03-14

**Authors:** Christian J Barton, Pazit Levinger, Kay M Crossley, Kate E Webster, Hylton B Menz

**Affiliations:** 1 School of Physiotherapy, Faculty of Health Sciences, La Trobe University, Bundoora, Victoria, Australia; 2 Musculoskeletal Research Centre, Faculty of Health Sciences, La Trobe University, Bundoora, Victoria, Australia; 3 Department of Mechanical Engineering, University of Melbourne, Victoria, Australia; 4 School of Physiotherapy, University of Melbourne, Victoria, Australia

## Abstract

**Background:**

Foot posture assessment is commonly undertaken in clinical practice for the evaluation of individuals with patellofemoral pain syndrome (PFPS), particularly when considering prescription of foot orthoses. However, the validity of static assessment to provide insight into dynamic function in individuals with PFPS is unclear. This study was designed to evaluate the extent to which a static foot posture measurement tool (the Foot Posture Index - FPI) can provide insight into kinematic variables associated with foot pronation during level walking in individuals with PFPS and asymptomatic controls.

**Methods:**

Twenty-six individuals (5 males, 21 females) with PFPS aged 25.1 ± 4.6 years and 20 control participants (4 males, 16 females) aged 23.4 ± 2.3 years were recruited into the study. Each participant underwent clinical evaluation of the FPI and kinematic analysis of the rearfoot and forefoot during walking using a three-dimensional motion analysis system. The association of the FPI score with rearfoot eversion, forefoot dorsiflexion, and forefoot abduction kinematic variables (magnitude, timing of peak and range of motion) were evaluated using partial correlation coefficient statistics with gait velocity entered as a covariate.

**Results:**

A more pronated foot type as measured by the FPI was associated with greater peak forefoot abduction (r = 0.502, p = 0.013) and earlier peak rearfoot eversion relative to the laboratory (r = -0.440, p = 0.031) in the PFPS group, and greater rearfoot eversion range of motion relative to the laboratory (r = 0.614, p = 0.009) in the control group.

**Conclusion:**

In both individuals with and without PFPS, there was fair to moderate association between the FPI and some parameters of dynamic foot function. Inconsistent findings between the PFPS and control groups indicate that pathology may play a role in the relationship between static foot posture and dynamic function. The fair association between pronated foot posture as indicated by the FPI and earlier peak rearfoot eversion relative to the laboratory observed exclusively in those with PFPS is consistent with the biomechanical model of PFPS development. However, prospective studies are required to determine whether this relationship is causal.

## Background

Foot posture assessment is frequently undertaken in clinical practice for the evaluation of individuals with lower limb overuse injuries, particularly when considering prescription of foot orthoses. One condition for which foot posture assessment is commonly performed is patellofemoral pain syndrome (PFPS) [1,2], as it is believed that individuals with PFPS who demonstrate signs of excessive foot pronation are likely to benefit from foot orthoses [1,2]. It is theorised that controlling excessive foot pronation will, in turn, limit the amount of tibial and femoral rotation; kinematic variables linked to patellofemoral joint loading [3-5].

Despite a paucity of empirical evidence supporting the theoretical rationale underpinning foot orthoses prescription for individuals with PFPS, most studies evaluating the foot orthoses efficacy in this population have only included individuals with signs of "excessive" pronation [6]. However, there is no consensus amongst these studies for the most valid method to evaluate foot pronation [6]. Additionally, the reliability and validity of previous methods used for individuals with PFPS have not been adequately examined [6]. Considering the emphasis on assessing foot pronation when prescribing foot orthoses for individuals with PFPS, valid, reliable and easy to implement clinical tests are essential. Razeghi and Batt [7] completed a critical review of clinically based foot classification and observed that many clinically based measures of foot posture possessed good reliability and face validity. However, they noted that the ability of foot posture assessments to predict dynamic function has not been well established [7].

One easy to implement clinical assessment tool to evaluate foot posture with good face validity is the Foot Posture Index (FPI) [8]. The FPI evaluates the multi-segmental nature of foot posture in all three planes and does not require the use of specialised equipment [8]. Additionally, our recent study indicated that the FPI was able to detect differences between those with and without PFPS (i.e. more pronated foot type in the PFPS group) and also possessed high intra- and inter-rater reliability individuals with PFPS (ICCs) [9]. Although this study provided some justification for the use of the FPI in clinical and research settings involving individuals with PFPS, its ability to provide insight into dynamic function in this population is unclear.

A number of studies attempting to correlate clinical measures of foot posture with dynamic foot function during gait in healthy individuals have been published [10-13] since Razeghi and Batt's [7] review. Although all of these studies reported static clinical measurements to be associated with dynamic function, a number of methodological issues need to be considered, particularly when attempting to apply these findings to a PFPS population. Three of these studies [10,11,13] used two dimensional video analysis, which may not provide adequate representation of the multiplanar three-dimensional motion occurring at the foot during gait. Additionally, one study evaluated arch height [13] which has subsequently been found to poorly discriminate between individuals with PFPS and controls [9]; and two [10,11] evaluated longitudinal arch angle, which exhibits poor reliability in individuals with PFPS [9]. Finally, all four studies [10-13] evaluated an asymptomatic population, limiting their applicability to a PFPS population.

In a recent study, Chuter [12] evaluated the relationship between three-dimensional rearfoot kinematics and the FPI and reported that the FPI score was able to explain 85% of the variance in peak rearfoot eversion. However, like other studies which evaluated clinical foot posture measures [10,11,13], these findings were limited to a population without defined pathology. Only one study has evaluated the association of static with dynamic foot function in individuals with PFPS [14]. Although this study reported that static relaxed calcaneal angle was able to explain 59% of the variance in peak rearfoot eversion [14], the three dimensional marker based analysis used for static assessment is not easily replicated in a clinical setting.

Considering the findings presented above, there appears to be a paucity of studies evaluating relationships between static foot posture and dynamic foot function, specifically in individuals with PFPS. The two studies evaluating three dimensional kinematics have included only one kinematic variable: the magnitude of peak rearfoot eversion [12,14]. Therefore, the effect of static foot posture on other kinematic parameters associated with foot pronation often observed visually in a clinical setting, such as forefoot dorsiflexion (arch flattening) and abduction, remains unclear. Additionally, the association of foot posture with kinematics previously linked to PFPS including peak rearfoot eversion timing [15-18] and range of motion [16] has not been previously evaluated.

Considering the good face validity and previously established reliability of the FPI in individuals with PFPS [9], this study was designed to further investigate its validity (i.e. ability to provide insight into dynamic function). Specifically, the degree of correlation between the FPI and (i) forefoot dorsiflexion; (ii) forefoot abduction, and (iii) rearfoot eversion kinematics during walking was evaluated in individuals with PFPS and asymptomatic controls.

## Methods

### Participants

Patellofemoral pain syndrome and control participants were recruited from a case-control study evaluating lower limb kinematics [18]. All participants were recruited via advertisements placed at La Trobe University, Melbourne University and on noticeboards in the greater Melbourne area. All participants gave written informed consent prior to participation and were recruited into the study over the same period of time. Ethical approval was granted by La Trobe University's Faculty of Health Sciences Human Ethics Committee. Participants included 26 individuals with PFPS (5 males and 21 females) and 20 asymptomatic controls (4 males and 16 females). Mean (SD) age, height and mass of the PFPS participants was 25.1 (4.6) years, 168.6 (8.4) cm, and 66.7 (12.8) kg respectively. Mean (SD) age, height and mass of the control participants was 23.4 (2.3) years, 171.1 (8.4) cm, and 66.0 (15.4) kg, respectively. The physical activity levels of participants from each group was measured using the long version of the 7 day self administered International Physical Activity Questionnaire (IPAQ) [19]. Mean (SD) weekly activity levels were 5801 (2991) and 4761 (3937) metabolic equivalents for the PFPS and control groups, respectively.

Diagnosis of PFPS was based on definitions used in previous RCTs [20,21]. Inclusion criteria were: aged 18 - 35 years old; insidious onset of peripatellar or retropatellar knee pain of at least 6 weeks duration; worst pain in the previous week of at least 30 mm on a 100 mm visual analogue scale; pain provoked by at least two activities from running, walking, hoping, squatting, stair negotiation, kneeling, or prolonged sitting; pain elicited by patellar palpation, PFJ compression or resisted isometric quadriceps contraction. Exclusion criteria were: concomitant injury or pain arising from the lumbar spine or hip; knee internal derangement; knee ligament insufficiency; previous knee surgery; PFJ instability; or patellar tendinopathy. As the same participants were also recruited for a foot orthoses clinical prediction rule study, additional exclusion criteria included use of foot orthoses in the previous five years. Control participants were required to be 18 - 35 years old, have no history of surgery or significant injury to the low back of lower limbs, have suffered no low back or lower limb pain in the previous six months which caused them to seek treatment or alter physical activity levels, and have not worn foot orthoses in the previous five years.

### Procedures

Each participant attended a single data collection session involving evaluation of the FPI and lower limb kinematics during walking. The tested limb used in the PFPS group was the symptomatic (in those with unilateral symptoms) or most symptomatic (in those with bilateral symptoms) limb. The tested limb in the control group was randomly selected to match the proportion of left and right limbs evaluated in the PFPS group. Prior to motion analysis testing, the FPI was recorded by a single rater with previously established intra-rater (ICC = 0.88 - 0.97) and inter-rater reliability (0.79 - 0.88) in a PFPS population [9].

Foot posture was evaluated using the FPI, a six item foot posture assessment tool, where each item is scored between -2 and +2 to give a sum total between -12 (highly supinated) and +12 (highly pronated) [8]. Items include: talar head palpation, curves above and below the lateral malleoli, calcaneal angle, talonavicular bulge, medial longitudinal arch, and forefoot to rearfoot alignment [8].

### Kinematic analysis

**Figure 1:**
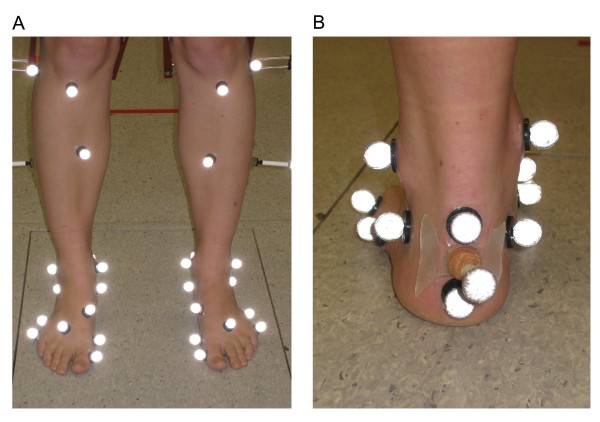
**Anterior view of Oxford foot model and plug-in-gait marker placements (A) and posterior view of Oxford foot model marker placements (B) for the static trial**.

Motion analysis was conducted using a three dimensional motion analysis system (Vicon MX system, Oxford Metrics Ltd, Oxford, England) with 10 cameras (8 × MX3 and 2 × MX40) operating at a sampling frequency of 100 Hz. Ground reaction forces were collected using two force plates (Kistler, type 9865B, Winterthur, Switzerland; and AMTI, OR6, USA) at a sampling frequency of 1000 Hz. Retro-reflective markers were placed on specific anatomical landmarks in accordance with the Oxford Foot Model (OFM) and PlugIn Gait as described by Stebbins et al [22] (see Figure 1). This allowed the formation of forefoot, rearfoot and tibial segments. The forefoot segment was formed by markers placed on the base of first metatarsal, head of first metatarsal, head of fifth metatarsal, and base of fifth metatarsal. The rearfoot segment was formed by three markers bisecting the heel (distal, wand, and proximal), and markers placed on the lateral calcaneus and sustentaculum tali. The tibial segment was formed by markers placed on the head of the fibula, tibial tuberosity, anterior border of tibia, lateral aspect of tibia (5 cm wand), and medial and lateral malleoli. Additionally, the knee joint centre calculated from PlugIn Gait was used to define the tibial segment in the OFM. The following additional marker placements were required for the PlugIn Gait model to form the thigh and hip segments: lateral aspect of the femur (5 cm wand), the anterior superior iliac spine, and the sacrum (see Figure 1).

A relaxed standing calibration trial was then captured with knee alignment devices (KADs) in situ. The knee joint centre calculated from this static trial was used to define the tibial segment in the OFM. Prior to the walking trials, the KADs and the calibration markers used to define segment axes were removed (medial malleoli, proximal heel, and first metatarsal head). Practice walking trials to allow familiarisation with the instrumentation and environment were then performed. Once participants were comfortable and walking with consistent velocity, motion analysis data collection commenced. Each participant was asked to walk at their natural comfortable speed across a 12 m walkway. Five successful trials (i.e. instrumented foot landed within the borders of the first force plate they traversed) were collected for each participant. Participants were not made aware of the force plates and their starting position was modified by the investigator to enhance the chances of a successful trial.

### Data processing

Each trial was reconstructed and the retro reflective markers identified and labelled within the Vicon Nexus software. Initial heel strike and toe off were defined using force platform data. The second heel strike (signalling the end of the gait cycle) was defined as the point where the movement trajectory of the ipsilateral heel wand marker became stationary. Data processing was completed by applying the OFM. Processed data were then exported to a purposely developed Microsoft Excel (Microsoft Corporation, Redmond, Washington, USA) template for analysis. Variables of interest included magnitude and timing of peak angles and ranges of motion during stance for:

(i) Rearfoot relative to the laboratory (floor) - eversion

(ii) Rearfoot relative to tibia - eversion

(iii) Forefoot relative to rearfoot - dorsiflexion and abduction

### Statistical analysis

Prior to statistical analysis the ordinal FPI data were converted into Rasch transformed scores to allow parametric analysis of interval data [23]. Partial correlations with gait velocity entered as a co-variate were calculated to determine the association between each of the FPI Rasch-transformed scores and kinematic measures during walking. Gait velocity was included as a co-variate during statistical analysis due to previous PFPS case control research indicating that some individuals with PFPS may reduce their gait velocity [24], and the reported effects this reduction can have on lower limb kinematics [25-27]. Based on previous recommendations [28], correlations from 0.00 to 0.25 were considered poor, 0.25 to 0.50 were considered fair, 0.50 to 0.75 were considered moderate to good, and 0.75 to 1.00 were considered excellent. All statistical calculations were completed using SPSS version 17.0 (SPSS Inc, Chicago, Illinois, USA).

## Results

### Participant characteristics

There were no significant differences between the groups for age (p = 0.116), height (p = 0.316), mass (p = 0.73), or weekly physical activity levels (p = 0.370). There was a trend toward a reduction in gait velocity for the PFPS compared to the control group (1.37 ± 0.13 m/s *versus *1.45 ± 0.16 m/s, p = 0.073). Foot Posture Index scores for the PFPS and control groups ranged from -1 to 10 and -1 to 6 respectively. The number of participants from both groups falling into each foot type categories defined by Redmond et al [8] can be found in Table 1.

**Table 1:** Number of participants from each group with foot types defined by the Foot Posture Index

	Highly supinated (-5 to -12)	Supinated (-1 to -4)	Normal (0 to +5)	Pronated (+6 to +9)	Highly pronated (+10 to +12)
PFPS group	0	2	15	8	1
Control group	0	1	18	1	0

### Association between foot posture measurements and foot kinematics

Correlations between the FPI and kinematic variables for both groups can be found in Table 2. A more pronated foot type as measured by the FPI was associated with greater peak forefoot abduction (r = 0.502, p = 0.013) and earlier peak rearfoot eversion relative to the laboratory (r = -0.440, p = 0.031) in the PFPS group, explaining 28 and 23% of variance, respectively. Additionally, a more pronated foot type as measured by the FPI was associated with greater rearfoot eversion range of motion relative to the laboratory in the control group (r = 0.614, p = 0.009), explaining 37% of variance.

**Table 2:** Correlations between the Foot Posture Index score and foot kinematics

	PFPS group		Control group	
	**r value**	**p value**	**r value**	**p value**

Magnitude of peak angles				
Rearfoot eversion relative to laboratory	0.300	0.155	0.214	0.410
Rearfoot eversion relative to tibia	0.167	0.435	0.230	0.374
Forefoot dorsiflexion	0.031	0.886	-0.188	0.470
Forefoot abduction	0.502*	0.013	0.168	0.520
Timing of peak angles				
Rearfoot eversion relative to laboratory	-0.440*	0.031	0.088	0.736
Rearfoot eversion relative to tibia	-0.052	0.811	0.082	0.755
Forefoot dorsiflexion	-0.172	0.420	-0.321	0.209
Forefoot abduction	0.239	0.260	-0.327	0.200
Range of motion				
Rearfoot eversion relative to laboratory	0.135	0.528	0.614**	0.009
Rearfoot eversion relative to tibia	0.026	0.903	-0.122	0.640
Forefoot dorsiflexion	-0.281	0.183	0.215	0.408
Forefoot abduction	-0.340	0.104	0.122	0.641

* p < 0.05

** p < 0.01

PFPS = patellofemoral pain syndrome

Positive value = more pronated foot as measured by the FPI associated with greater peak magnitude, delayed peak timing and greater range of motion.

## Discussion

Foot posture is frequently evaluated in individuals with PFPS, particularly when considering prescription of foot orthoses. Evaluation of foot posture is often performed under the assumption that measuring static structure will provide insight into dynamic function, although this is largely unproven [7]. The current study is the first to evaluate the relationship between a clinical measure of foot posture with established reliability (the FPI) in individuals with PFPS [9] and three-dimensional kinematics associated with foot pronation.

In the current study, a more pronated foot, as indicated by the FPI, demonstrated fair association with earlier timing of peak rearfoot eversion relative to the laboratory during walking in the PFPS group. This finding is consistent with other recent findings by our group. In separate cohorts we found that individuals with PFPS possessed both earlier peak rearfoot eversion during walking [18], and a more pronated foot as measured by the FPI [9]. This indicates that earlier peak rearfoot eversion relative to the laboratory may be in part due to foot structure in individuals with PFPS. Considering this association did not occur in the control group, the relationship may be of particular significance to the development of PFPS. This may indicate that a more pronated foot posture results in more rapid dynamic foot pronation in people who are predisposed to PFPS development. Prospective studies are required to determine if this relationship is causal.

When measured relative to the tibia, rearfoot eversion timing differences during gait have been consistently reported in previous PFPS case control studies [15-18]. However, unlike kinematic measurement relative to the laboratory, the FPI did not provide insight into peak rearfoot eversion timing relative to the tibia during walking in either group. A possible explanation for the inconsistent findings between the two methods of rearfoot kinematic evaluation for the PFPS group may be the influence of tibial structure and function. When broken down, the majority of the six FPI components evaluate solely foot structure, with the exception of curves above and below the lateral malleolli. Additionally, two of these measures directly evaluate the rearfoot: talar head palpation and calcaneal angle. Therefore, a relationship with rearfoot motion relative to the lab may be expected. Conversely, none of the FPI components evaluates tibial structure, indicating a relationship may be less likely.

The presence of symptoms may partly explain the different associations between static and dynamic foot function found in individuals with PFPS compared to controls. However, an alternative explanation may be the presence of greater variation in foot posture for the PFPS group. The FPI scores for the PFPS group ranged from -1 to 10, with nine out of the 26 participants considered to possess a pronated foot type (>+5) [8]. However, in the control group, FPI scores ranged only from -1 to 6, with just one out of 20 participants considered to possess a pronated foot type. This lower variation in the control group will reduce the likelihood of finding a statistically significant association between the two variables [28].

Despite having less variation in foot posture, relationships between the FPI and kinematics not evident in the PFPS group were identified in the control group. A more pronated foot as measured by the FPI was moderately associated with greater rearfoot eversion range of motion relative to the laboratory. Interestingly, none of the three significant findings in this study were consistent between the two groups. Without prospective evaluation, it cannot be determined which of these relationships are causes and which are effects in relation to PFPS. However, they do highlight the need for caution when interpreting results based on asymptomatic populations. Results from the current study indicate that previous and future correlations identified when evaluating only asymptomatic populations may not exist in patients with PFPS.

Foot posture is often evaluated based on the assumption that it will provide insight into the magnitude of foot pronation during gait [7]. The FPI was recently found to possess good reliability and ability to discriminate between individuals with PFPS and controls [9]. However, findings from the current study indicate that insight into dynamic function from assessing the FPI may be limited to moderate and fair associations with peak forefoot abduction and timing of rearfoot eversion, respectively, in the PFPS group. Neither peak forefoot dorsiflexion, nor peak rearfoot eversion was associated with the FPI in either group, implying its utility in guiding clinical decisions when considering foot orthoses to control rearfoot eversion or forefoot dorsiflexion magnitude may be limited. Considering this, development of a reliable and easy to implement clinical assessment tool to evaluate dynamic foot function may be needed. This could potentially replace current static foot posture evaluation and provide greater guidance when considering foot orthoses prescription for individuals with PFPS.

Increased magnitude of peak rearfoot eversion during gait has been commonly considered as a potential contributor to PFPS [2,29]. However, previous case control findings indicate that greater peak rearfoot eversion is not present in individuals with PFPS during gait [15-18]. Additionally, findings from this study imply that a more pronated foot posture may not relate to PFPS pathology through influences on peak rearfoot eversion. Interestingly, in this study we found associations between the FPI and earlier peak rearfoot eversion relative to the laboratory, a kinematic feature we recently found to be associated with PFPS [18]. It is also possible that other biomechanical variables during gait previously linked to PFPS including knee [30] and PFJ [31,32] loading, and lower limb neuromuscular control [33-35] may be associated with foot posture. Investigating these possibilities may improve foot orthoses design for individuals with PFPS.

Contrary to findings in the current study, Chuter [12] recently reported that a more pronated foot as measured by the FPI was associated with greater peak rearfoot eversion in a group of participants without defined pathology. Although the effect of pathology on kinematics may explain equivocal findings between Chuter's [12] study and the PFPS group in the current study, such an effect cannot explain equivocal findings with the control group from the current study. However, there are two possible explanations for this disparity. Firstly, Chuter [12] evaluated a larger cohort (n = 40) than the two cohorts evaluated in the current study (PFPS = 26 and control = 20), which is likely to lead to stronger statistical associations between two variables [28]. Secondly, Chuter [12] selectively recruited a range of foot postures (i.e. 20 normal and 20 pronated foot types as measured by the FPI), while the current study recruited participants based on PFPS diagnosis and matched these with participants of similar ages, heights and body masses to form the control group. As a result, the spread of FPI scores was lower in the current study's control group which can also result in weaker statistical associations [28].

The results of this study need to be considered in the context of several limitations. Firstly, we chose to evaluate the FPI in this study based on the ease of clinical application, wealth of information provided, previous research establishing reliability [9] and strong face validity. However, other measures of foot posture such as radiographical evaluation may provide greater insight dynamic foot function in individuals with PFPS. Secondly, this study evaluated only rearfoot and forefoot kinematics based on the OFM. The OFM assumes that motion between these segments is transmitted through the midfoot [36]. Future studies may find additional correlations between static and dynamic foot function by using kinematic models which directly evaluate midfoot function. Thirdly, this study evaluated only one functional task, walking. Considering that pain may not be present during walking in all individuals with PFPS, future research should consider evaluating more strenuous tasks such as stair negotiation, squatting and running. Finally, the results of this study are based on retrospective case control evaluation. Therefore, whether inconsistent relationships found between the two groups are a cause or effect in relation to PFPS is unclear. Future prospective research evaluating the presence of any relationships between foot posture and function in those who develop PFPS is required.

## Conclusion

This is the first study to evaluate the relationship between foot posture and three-dimensional kinematics in individuals with PFPS. A more pronated foot as measured by the FPI was moderately associated with greater peak forefoot abduction and fairly associated with earlier peak rearfoot eversion relative to the laboratory in the PFPS group, and greater rearfoot eversion range of motion relative to the laboratory in the control group. Inconsistent findings between the PFPS and control groups indicate that pathology may play a role in the relationship between static foot posture and dynamic function. The association between pronated foot posture and earlier peak rearfoot eversion relative to the laboratory observed exclusively in those with PFPS is consistent with the biomechanical model of PFPS development. However, prospective studies are required to determine whether this relationship is causal.

## Competing interests

HBM is Editor-in-Chief of the *Journal of Foot and Ankle Research*. It is journal policy that editors are removed from the peer review and editorial decision making processes for papers they have coauthored.

## Authors' contributions

CJB coordinated all data collection and analysis. All authors were involved in the design of the study, interpretation of the results, helped draft the manuscript, and read and approved the final manuscript.
